# Automated generation of genome-scale metabolic draft reconstructions based on KEGG

**DOI:** 10.1186/s12859-018-2472-z

**Published:** 2018-12-04

**Authors:** Emil Karlsen, Christian Schulz, Eivind Almaas

**Affiliations:** 1 Department of Biotechnology and Food Science, NTNU - Norwegian University of Science and Technology, Høgskoleringen 1, Trondheim, 7491 Norway; 2 K.G. Jebsen Center for Genetic Epidemiology, Department of Public Health and General Practice, NTNU - Norwegian University of Science and Technology, Trondheim, Norway

**Keywords:** AutoKEGGRec, Genome-scale metabolic model, Constraint-based analysis, Pipeline, Draft model, Metabolic network reconstruction, Multiple organisms, Community model, Consolidated model, COBRA

## Abstract

**Background:**

Constraint-based modeling is a widely used and powerful methodology to assess the metabolic phenotypes and capabilities of an organism. The starting point and cornerstone of all such modeling is a genome-scale metabolic network reconstruction. The creation, further development, and application of such networks is a growing field of research thanks to a plethora of readily accessible computational tools. While the majority of studies are focused on single-species analyses, typically of a microbe, the computational study of communities of organisms is gaining attention. Similarly, reconstructions that are unified for a multi-cellular organism have gained in popularity. Consequently, the rapid generation of genome-scale metabolic reconstructed networks is crucial. While multiple web-based or stand-alone tools are available for automated network reconstruction, there is, however, currently no publicly available tool that allows the swift assembly of draft reconstructions of community metabolic networks and consolidated metabolic networks for a specified list of organisms.

**Results:**

Here, we present AutoKEGGRec, an automated tool that creates first draft metabolic network reconstructions of single organisms, community reconstructions based on a list of organisms, and finally a consolidated reconstruction for a list of organisms or strains. AutoKEGGRec is developed in Matlab and works seamlessly with the COBRA Toolbox v3, and it is based on only using the KEGG database as external input. The generated first draft reconstructions are stored in SBML files and consist of all reactions for a KEGG organism ID and corresponding linked genes. This provides a comprehensive starting point for further refinement and curation using the host of COBRA toolbox functions or other preferred tools. Through the data structures created, the tool also facilitates a comparative analysis of metabolic content in any given number of organisms present in the KEGG database.

**Conclusion:**

AutoKEGGRec provides a first step in a metabolic network reconstruction process, filling a gap for tools creating community and consolidated metabolic networks. Based only on KEGG data as external input, the generated reconstructions consist of data with a directly traceable foundation and pedigree. With AutoKEGGRec, this kind of modeling is made accessible to a wider part of the genome-scale metabolic analysis community.

**Electronic supplementary material:**

The online version of this article (10.1186/s12859-018-2472-z) contains supplementary material, which is available to authorized users.

## Background

Genome-scale metabolic modeling has gained a sharp increase in popularity in recent years. Currently, there exist more than 160 well curated metabolic network reconstructions [1] spanning more than 100 species, such as the Bacteria *Bacillus, Clostridium, Escherichia, Pseudomonas*, and *Salmonella*, the Eukaryota *Homo sapiens*, and *Saccharomyces*, and the Archaea *Methanosarcina*, to name just a few. While many of these reconstructions were created years ago, they are still a helpful and reliable source when constructing a new genome-scale metabolic model (GEM) for another species, or to extend the knowledge of an existing reconstruction. However, the metabolic networks are often reconstructed using quite different methodologies and approaches with a wide variety of annotation conventions. Consequently, the comparison of genome-scale reconstructions is not straightforward when the interest is in identifying how much a set of reconstructed networks actually differ in biological content. This challenge needs to be addressed by the modeling community in order to avoid unnecessary repetition of arduous reconstruction work [2, 3].

For manipulating and curating these model metabolic networks, different tools exist. Most notable is the COBRA Toolbox [4], an open-source community-driven software suite available in Matlab. This toolbox contains a large number of built-in functions for the analysis and refinement of constraint-based metabolic models. Having found numerous and potent applications within modeling and analysis in fields such as biology and medicine, it remains a popular and well-supported platform for such work [4, 5]. However, it currently contains no tools for the rapid assembly of large-scale first-draft models.

The development of tools to aid and assist in the reconstruction of GEMs has recently been an area of active research [6–15]. For the fully automated creation of models, tools such as PathwayTools [6], ModelSEED [7], SuBliMinaL [8], FAME [10], RAVEN [12], CoReCo [13], Merlin [14], PyFBA [15], and CarveME [16] are intended to generate a fully functioning first draft reconstruction (FDR), or even first draft model, based on core skeleton template models and/or whole-genome sequence annotations of the organism in question. In-depth comparisons of the capabilities of these tools is available elsewhere [17] and within the original papers presenting the tools.

Some of the tools, such as ModelSeed [7], MicrobesFlux [11], and FAME [10] are web-based tools, whereas others might necessitate a download of databases that they rely on or a list of required additional software.

Of the tools mentioned above, CarveMe [16] has a notably different and novel approach: From a manually curated universal template model, a reconstruction for a particular organism is “carved” using a reaction reduction process based on the genome annotation for the chosen organism [16]. At all times during this reaction-reduction process from the template, the shrinking metabolic network is under the constraint of having to support a specified biomass function. This rapid reconstruction process makes it well suited in situations where a large number of FDRs must be generated rapidly, such as for the modeling of microbial communities, and indeed the tool automates the process of merging single-organism metabolic networks into community-level networks [16].

MEMOSys [9] is a stand alone tool, mainly created for managing, developing and storing GEMs but not primarily for generating them.

The aforementioned reconstruction tools have various strengths, thus offering the modeling community a strong base to create and curate single organism FDRs suitable to a user’s needs. In addition to these, more specialized tools exist, such as KEGG2SBML [18], which translates KEGG Metabolic Pathway files into SBML.

However, as is well known within the field, even when using one of the tools capable of automatically generating FDRs, it is a necessity to use a host of other tools or software packages in a manual-curation process to optimize a reconstruction’s prediction accuracy [7]: It has been observed that, while automatically generated first draft GEMs may be capable of producing biomass, this ability may not be reflected in a GEM’s accuracy in predicting experimental outcomes [19]. Thus, much manual curation work is required in order to refine and optimize a model. Such manual curation steps may include gap-filling (the addition of reactions inferred from the flow of metabolites through the metabolic network), the correction of reactions that are improperly mass or charge balanced (which could result in the spontaneous generation of mass or energy within the system), or the modification of reactions that are thermodynamically infeasible.

In general, it is also necessary to manually add and adjust transport reactions, their presence automatically surmised from experimental evidence of uptake rates, cytosolic activity, or template reconstructions. Finally, the biomass composition function is notoriously difficult to assess correctly, as the biomass composition might be subtly different for two related strains in the same medium, but drastically different for the same strain in two different media [20]. While the maximization of biomass production is a much used primary cellular objective in genome-scale metabolic modeling, this is not necessarily the case in vivo, and therefore deciding on a reasonable cellular objective for a given organism may be far more convoluted than simply finding its biomass composition [20].

There is an increasing interest in the modeling of microbial communities, both due to the fact that it is becoming more and more tractable from a technical standpoint, and because of the mounting evidence for the importance of microbiomes in ecology [21], industry [22, 23], and health [24, 25]. In light of this, it seems that for any application outside of highly controlled environments where a monoculture may be maintained, such as in the lab or in industrial bioreactors, capturing the dynamics of interactions between different populations of microbes is essential to understand the behavior of almost any biological system.

However, constructing GEMs for a microbial community is an arduous and time-consuming process: It is not simply a case of twice the organisms resulting in twice the work. As the number of organisms increases, the complexity of the potential interactions also increases exponentially. In order to save time and effort to make the task more tractable, it is therefore imperative to supply researchers with a set of tools that allow the efficient automation of, at least, parts of this reconstruction process. This is a topic of research and development receiving much attention at current conferences.

As a contribution to fill this capability gap, we created a pipeline within the COBRA Toolbox in Matlab named AutoKEGGRec that only uses the Kyoto Encyclopedia of Genes and Genomes (KEGG) database [26–28] as external input. AutoKEGGRec assembles a draft reconstruction for any given query list of KEGG organisms. Basing itself solely on annotations present in this extensive and actively maintained database rather than on template models, potential historic biases from previous reconstructions (positive or negative) are eliminated. Only reactions known and documented in KEGG to be present in the organism are used within the first draft reconstruction. Adding this function to the COBRA toolbox, the most widely used software suite for constraint-based modeling of GEMs, has as a consequence that no further software installation is required for additional metabolic network curation, and that the scientific community may alter or update the AutoKEGGRec functionality as they see fit.

AutoKEGGRec is easy to use, and there is no need for conversion of model files to work with supported SBML versions and requirements of the COBRA toolbox. Specifically, AutoKEGGRec allows the construction of a large number of draft reconstruction networks, as well as the direct assembly of community reconstructions. In the process of assembling these reconstructions, the AutoKEGGRec function also constructs a consolidated draft metabolic network consisting of a union of all metabolic reactions in the query organisms. Note that analyses may be performed on the consolidated network and other data structures generated by the function.

## Implementation

AutoKEGGRec is designed in Matlab 2017b, hereafter referred to as Matlab, and uses functions within the COBRA Toolbox v3.0, hereafter referred to as COBRA toolbox/COBRA. Since AutoKEGGRec uses existing COBRA toolbox functions for saving the models, the generated data structures containing the reconstructed model work seamlessly with all COBRA functions. In the following, we will illustrate functions of AutoKEGGRec by using a set of query organisms consisting of the five *E. coli* K-12 strains with the KEGG IDs *eco*, *ecj*, *ecd*, *ebw*, and *ecok* and explain the overall design of the pipeline. A user Manual [Additional file 1] and a matlab version of the tool [Additional file 2] are provided together with the article. AutoKEGGRec will be submitted to be integrated with the COBRA Toolbox.

### Overall design

**Fig. 1:**
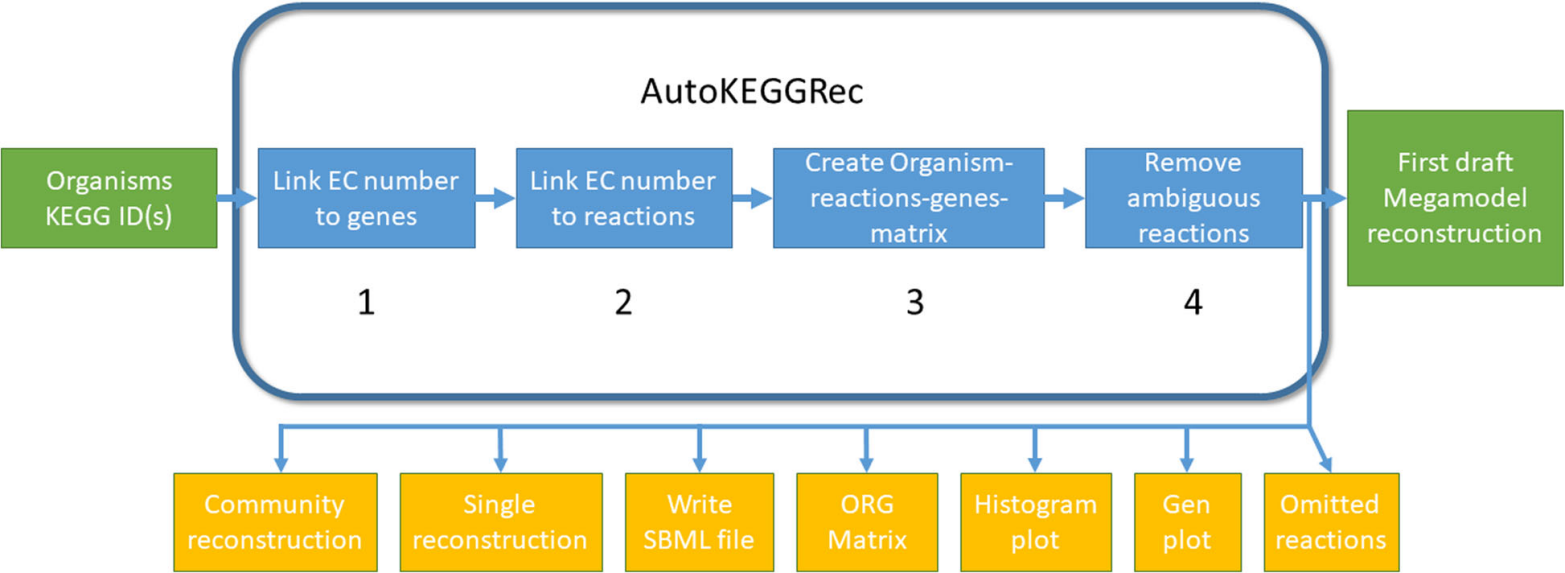
General workflow of the AutoKEGGRec pipeline. KEGG organism ID(s) are needed as input to the function (leftmost green box), and optional flags may be set (yellow boxes). Using KEGG IDs to fetch relevant data from the KEGG database and handle them within the pipeline, (1) all links between EC numbers and their genes and (2) the further linkage between EC numbers and reactions (2) are stored. From this information, (3) an Organisms-Reactions-Genes matrix is constructed. (4) Reactions are filtered by e.g. removing polymerization or generic reactions. A first draft consolidated model reconstruction is returned (rightmost green box)

Designed to be a fast tool that is easy to use, AutoKEGGRec facilitates the rapid assembly of draft models from KEGG annotations for one or several query organisms. Internally, it’s designed for efficiency of operation, first minimizing the number of queries sent to the KEGG servers during data collection stage and, subject to this, minimizing the number of operations needed to assemble the draft model from the collected data. Intended to fit into the COBRA toolbox as a function, AutoKEGGRec requires no further installation or setup. While not necessary for its intended operation, it is directly amenable to be modified by each user as required since it is provided as a Matlab function. Figure 1 gives a graphical overview of the different steps involved in the creation of the first draft reconstruction (right green box), and it highlights the additional functionality available using the optional flags (yellow boxes).

AutoKEGGRec needs the input of KEGG organism IDs for the user-chosen organisms as a list of strings followed by the optional flags discussed further in the Manual (leftmost green box in Fig. 1). The pipeline initiates by using the KEGG organism IDs for querying the linkage between enzyme commission (EC) numbers and the corresponding genes from KEGG for each organism, as per step (1) in Fig. 1. In step (2), AutoKEGGRec retrieves the linkage between reactions and EC numbers. Based on the assembled data, the function generates matrices that use EC numbers to store the linkage between KEGG reaction ID and the genes of the organisms. Next, every reaction in KEGG associated with one or more query organisms is retrieved and related to the target organism(s) via corresponding genes (step (3)). This Organisms-Reactions-Genes (ORG) matrix is returned by AutoKEGGRec when submitting the *OrgRxnGen*-flag (yellow box in Fig. 1), and is thus directly available for further analysis by the user. At this stage, each reaction together with its complete annotation is downloaded from KEGG, as well as the corresponding compound annotations. Since the single organism models and the community model can both be generated more rapidly using the consolidated model, the latter model is generated by default.

Note that the steps involved in downloading all relevant information from KEGG is generally the most time consuming part of the pipeline, except for the case of large community models consisting of roughly 15 or more organisms, refer to Table 2. Consequently, we have implemented the download process using the Matlab function *parfor* for parallelization; in anticipation of this, the parpool functionality is initiated by default at the beginning of the function execution and shut down in the end.

All the reactions in the draft metabolic network(s) are filtered to avoid redundant or poorly defined reactions (step (4)). For example, if the “Comment”-field in KEGG contains “Remark” and the reaction equation contains *G* (glycan instead of a compound name) or *n* (polymer, generic reaction), the reactions are not included in the reconstruction. If the “Remark” field contains the strings “Generic”, “General”, or “General reaction”, these reactions are also omitted. Additionally, the compound information stored within KEGG occasionally note whether a compound is generic, and lists exact mass. When mass is set to 0 for a compound, this signifies it as a generic compound, and reactions that contain it are omitted from the reconstruction.

Omitted reactions are, for the sake of transparency and potential utility, stored within an additional Matlab structure, which is accessible for the user using the *OmittedData* option flag. These reactions are marked with a new *rxnAttention* field which explains why AutoKEGGRec omitted them from the reconstruction. This allows the user to first identify omitted reactions relevant to their project, followed by a curation step before they are potentially included.

In generating an FDR, AutoKEGGRec will add the complete reaction annotations: where possible, annotation fields supported by COBRA are used, but some new fields are, by necessity, created to store the information. Using the *createModel* function in the COBRA toolbox, the consolidated-type draft reaction network is created based on KEGG genome annotation and linked reactions. The GPR (gene-protein-reaction) rules are added based on the ORG matrix, and the model is annotated according to the KEGG information for the reactions and components. This includes compound names, reaction names, and compound KEGG IDs, to name just a few.

AutoKEGGRec will generate individual draft network reconstructions for each of the listed organisms when the *SingleModels* option flag is provided. These draft models are generated by first making multiple copies of the consolidated model (one for each organism), before removing reactions not specifically present in each organism. The community model is generated using the COBRA *createModel* function with the complete list of reactions as input. Here, each organism appears as a separate compartment since AutoKEGGRec will add the organism’s KEGG ID to all of its compounds.

Since the resulting output is a Matlab structure variable, the user only needs to define a single output variable independent of the number or combination of optional input flags.

### AutoKEGGRec optional settings

In Fig. 1, the separate option flags are indicated by the presence of yellow boxes, one for each optional setting. A discussion and description of the specific functionality provided by the different options is to be found within the Manual. Note that, AutoKEGGRec will create a consolidated reconstruction if no option flags are passed along with the function call as this is the default option. The option flags *OrgRxnGen*, *GenePlot* and *Histogram* may be used without generating a reconstruction, only providing the information listed for these flags (corresponding to steps 1-3 in Fig. 1). In this case, annotations for individual reactions and compounds are not downloaded, allowing AutoKEGGRec to execute significantly faster. Using the option flag *OmittedData* requires the download of all target reactions from KEGG, but no reconstruction is generated or stored, unless a flag to build one is passed.

### Main matrices - Organisms-Reactions-Genes matrix

The internal workings of AutoKEGGRec rely on a series of arrays and matrices, leveraging Matlab’s efficient handling of these data structures. All reactions in KEGG are compiled into an ordered list. To facilitate the fast conversion from KEGG ID back to internal reaction indexing, a reference table is constructed. All genes associated with the organisms in the user-query are fetched from KEGG along with their associations with EC numbers, the full set of linkages between EC numbers, and the reactions they catalyze. In the same way as for the reactions, reference lookups from string IDs to internal list indexes are built, allowing swift translation between these two identifiers.

From these index listings, several binary matrices are constructed. One matrix matches EC numbers to the reactions. Based on this matrix, AutoKEGGRec constructs the Organisms-Reactions (OR) matrix, that matches organisms to reactions. Thus, the OR matrix describes a bipartite graph representing the reactome of each query organism as given by KEGG from the OR matrix, a complete list of every reaction present in at least one of the query organisms is assembled, allowing further KEGG queries to be conducted only for these reactions. Also, AutoKEGGRec constructs the Organisms-Reactions-Genes (ORG) matrix using data in the OR matrix.

Table 1 shows a subsection of the ORG matrix for *E. coli* K-12 to illustrate the organization of information. Here, each row corresponds to a KEGG reaction ID, and empty rows indicate that the particular reaction ID is not present in any of the organisms (contains a zero entry in the columns Sum, Total, and Genes). In this example, a value of five in the Sum column and a value of unity in the Total column shows that the reaction is present in all *E. coli* K-12 strains. The genes associated with each reaction in the organisms are separated with, an “OR”-relationship. The number of genes per reaction is summarized in the “Genes” column; if the value varies between the organisms, a list of the different values are reported.

**Table 1:** Example output using the *OrgRxnGen* flag within AutoKEGGRec

KEGG ID	eco	ecj	ecd	ebw	ecok	Sum	Total	Genes
R00001						0	0	0
R00002						0	0	0
R00004	b4226	JW4185	ECDH10B_4421	BWG_3936	ECMDS42_3668	5	1	1
R00005						0	0	0
R00006	b0078, b3670, b0077, b3671, b3769	JW0077, JW3645, JW0076, JW3646, JW3742	ECDH10B_3853, ECDH10B_3958, ECDH10B_3854	BWG_0073, BWG_0074, BWG_3454, BWG_3361, BWG_3362	ECMDS42_3207, ECMDS42_0071, ECMDS42_0072, ECMDS42_3105, ECMDS42_3106	5	1	3;5
R00008						0	0	0
R00009	b1732, b3942	JW1721, JW3914	ECDH10B_1870, ECDH10B_4131	BWG_1545, BWG_3611	ECMDS42_1407, ECMDS42_3380	5	1	2
R00010	b1197, b3519	JW3487, JW1186	ECDH10B_3696, ECDH10B_1250	BWG_3208, BWG_1022	ECMDS42_2954, ECMDS42_0984	5	1	2
R00011						0	0	0
R00012						0	0	0
R00013	b0507	JW0495	ECDH10B_0463	BWG_0384	ECMDS42_0400	5	1	1
R00014	b0114, b0078, b3670, b0077, b3671, b3769	JW0110, JW0077, JW3645, JW0076, JW3646, JW3742	ECDH10B_0094, ECDH10B_3853, ECDH10B_3958, ECDH10B_3854	BWG_0073, BWG_0074, BWG_3454, BWG_3361, BWG_3362, BWG_0107	ECMDS42_3207, ECMDS42_0071, ECMDS42_0072, ECMDS42_0105, ECMDS42_3105, ECMDS42_3106	5	1	4;6
R00015						0	0	0
R00017	b3518	JW3486	ECDH10B_3695	BWG_3207	ECMDS42_2953	5	1	1

Here the Organism-Reaction-Gene matrix for the five *E. coli* K-12 strains is shown. The genes encoding the the first 14 reactions in KEGG, followed by the genes, if any, for the different organisms. Also provided with this output, in the three rightmost columns, is the **Sum** of organisms whose metabolism contains this reaction, the **Total** fraction of strains whose metabolism contains this reaction, and the number of **Genes** for the different organisms for that reaction

### Critical issues

Note that AutoKEGGRec is not intended to produce a fully functioning model, but a first draft reconstruction based on the KEGG database through a set of simple and transparent procedures. There are a few things to keep in mind:

- The pipeline uses only KEGG organism IDs as input.
- Glycan reactions are mostly removed. Since some of the glycans also have compound numbers within KEGG, these are still included in the first draft reconstruction. Reactions containing only glycans, i.e. KEGG metabolite IDs starting with *G* instead of *C*, are not included in the reconstruction even if there is no alternative reaction.
- Polymer reactions or non-specific reactions dealing with an of amount *n* or *m* of a compound *C* are not included by AutoKEGGRec: The constraint-based modeling is based on a steady-state or semi-steady-state approximation, and there is no consensus in the field for how one handles polymerization reactions. The user may edit these reactions to allow their inclusion in a reconstruction.
- There is no assignment of reactions to different compartments. All reactions and compounds are placed in the default compartment “cytosol” by the COBRA function *createModel*. Even though KEGG includes a few transport reactions for some organisms, these are not explicitly marked.
- Genes are linked to reactions. In the case of more than one gene associated with a reaction, the genes are linked together with “OR”-relationships since KEGG does not specify how the gene-products interact. Consequently, the user must manually curate these relations, providing the correct “AND” or “OR” relations between genes.
- Reactions with massless compounds are omitted. This step is necessary to maintain a mass balance within the reconstruction. Some of these reactions contain compounds like “protein” (*C*00017) or “DNA” (*C*00039), but also more specific compounds like “DNA adenine” (*C*00821).

## Results

Our implementation of an FDR pipeline fully integrated with the much used COBRA toolbox for Matlab makes it an easily accessible resource for the constraint-based modelling community. By basing the reconstruction of the FDRs solely on the KEGG database, the results reflect the quantity and quality of the KEGG curation, which is actively updated and maintained by the community. Note that, because of the lack of directionality of annotations in KEGG, all reactions are reversible in the FDR. In most cases, this choice will not coincide with biological reality; however, there exists COBRA functions to estimate the reaction directionality according to thermodynamics [29, 30].

Reactions are mass and charge balanced according to the KEGG reaction annotations, which are generally of high quality. We note that the manual determination of e.g. biomass reaction, transport reactions, and reaction directionality, are all expected steps in the process of transforming an automatically generated FDR to a high-quality curation. With AutoKEGGRec, the source and assembly of the metabolic network data is clear and all contained in the KEGG namespace, forming a solid foundation upon which to refine and curate the model, making it a well-suited alternative for proficient COBRA toolbox users.

**Fig. 2:**
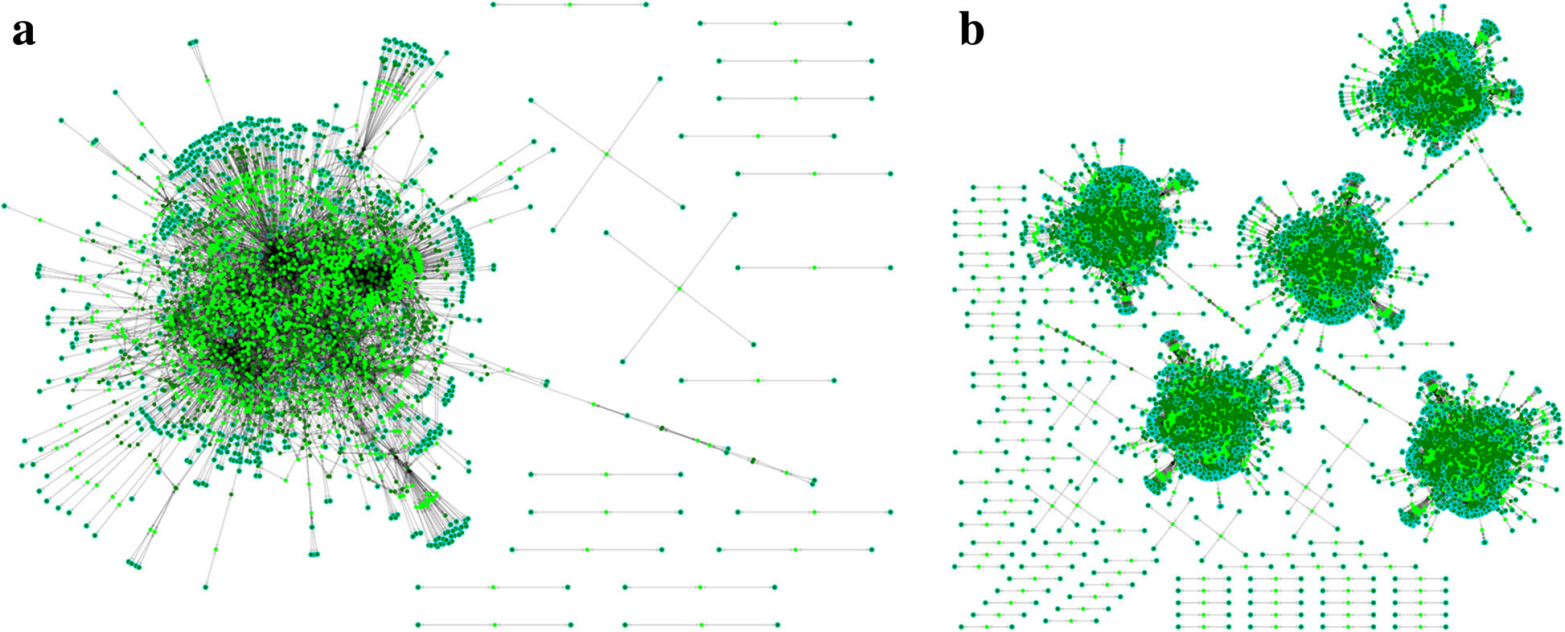
Consolidated FDR (**a**) and community FDR (**b**) generated from five *E. coli* K-12 strains. The consolidated FDR (**a**) consists of the union of metabolic reactions for the query organisms. The displayed network consists of 1596 reactions (light green) and 1621 metabolites (dark green), and is based on the five *E. coli* K-12 strains with KEGG organism IDs *eco*, *ecj*, *ecd*, *ebw*, and *ecok*. The vast majority of metabolic reactions can be seen to reside in the giant component. The community metabolic network (**b**) generated by AutoKEGGRec keeps the query organisms in separate compartments. The network consists of 8002 metabolites (dark green) and 7855 reactions (light green), with the vast majority of reactions associated with the five largest connected components. Note, that the different organisms are not connected due to the fact, that this consolidated FDR does not contain transport reactions, which would connect the different organisms/compartments

In Table 2, the timings and sizes of the giant components for a range of test examples are listed. We note that the percentage of all reactions found as part of the giant component for the various draft reconstructions is close to 99*%*; the resulting FDRs are graphically represented in Fig. 2 a and b. This speaks to the quality of the KEGG database and its annotations and to the promise of FDR assembly from high-quality curated databases.

The runtime of AutoKEGGRec is, for most applications, dominated by the reaction and compound retrieval-time from KEGG (seen in Table 2).

**Table 2:** Average execution time for AutoKEGGRec within Matlab 2017b on a Dell Latitude 7490 with an I7-7600U using 2-core-parallelization

Tested strains incl. number of organisms	Mean runtime [s]	Reactions/metabolites	Giant component [#, %]
*E. coli* K-12, 5 KEGG IDs	2,995±65	1226/1188	1215/99.10*%*
*P. putida*, 12 KEGG IDs	3,957±166	1412/1445	1400/99.15*%*
*B. subtilis*, 15 KEGG IDs	3,538±216	1108/1134	1103/99.55*%*
*E. coli*, 20 KEGG IDs	4,792±289	1342/1302	1331/99.18*%*
*E. coli*, 25 KEGG IDs	5,618±322	1351/1312	1340/99.19*%*
*E. coli*, 30 KEGG IDs	6,724±130	1354/1316	1343/99.19*%*
*E. coli*, 35 KEGG IDs	7,750±208	1358/1319	1347/99.19*%*
*E. coli*, 40 KEGG IDs	9,115±198	1359/1321	1348/99.19*%*
*E. coli*, 45 KEGG IDs	10,032±651	1359/1321	1348/99.19*%*
*E. coli*, 50 KEGG IDs	11,466±1,065	1359/1321	1348/99.19*%*
*E. coli*, 55 KEGG IDs	12,687±1,269	1359/1321	1348/99.19*%*
*E. coli*, 60 KEGG IDs	13,848±1,658	1359/1321	1348/99.19*%*
*E. coli*, 65 KEGG IDs	15,082±1,850	1360/1323	1349/99.19*%*

The columns are: a specification of the queried organisms, the mean and standard deviation runtime (five separate software executions) per dataset using *ConsolidatedRec*, *SingleRecs*, *CommunityRec*, *OrgRxnGen*, *OmittedData* and *DisconnectedReactions* as optional flags, the number of reactions and metabolites in the consolidated FDR and the number and fraction of reactions in the consolidated network’s giant component

The number of omitted reactions for the five mentioned *E. coli* K-12 strains is 519. Of these, 141, about 8.08*%* of the 1745 reactions associated with all the *E. coli* K-12 strain genes in KEGG, were removed based on reaction filtering. Of these, 48 were generic reactions, 6 are polymerization reactions, and 53 include glycans and are therefore not stored in the FDR. In the latter case, alternative reactions containing the KEGG IDs compound names are stored within the FDR if they exist in KEGG. A total of 31 reactions were removed from the FDR for being ill-defined (containing the same compound on both sites of the reaction equation), and 3 reactions were excluded from the FDR because of potentially being polymerization reactions. AutoKEGGRec evaluates the 1626 compounds of the remaining 1604 reactions, to identify generic (mass-less) compounds. In the example case of the *E. coli* K-12 strains this is true for 390 compounds, which is about 23.98*%*. These compounds participate in 378 reactions, which increases the number of omitted reactions to 519 (29.74*%*), and leaves the FDR with 1226 high quality reactions. All the mentioned reactions are detailed in the output data structure generated by the *omittedData* option flag, the omitted compounds and the saved KEGG annotations are stored as well, examples of outputs of the omitted data are shown in the Manual.

As an example use of our proposed model field “RxnAttention”, 10 reactions were marked in the generated FDR. One such reaction is

$$ R05994: G00124 + C00001 \Leftrightarrow G00123 + C00124, $$

which has two glycan compounds. In this case, these compounds correspond to the “normal” KEGG compound IDs *C*06136 and *C*06135, leading to an alternative reaction in the KEGG reaction universe using only these *C* compound names as

$$ R05112: C06136 + C00001 \Leftrightarrow C06135 + C00124. $$

Here, the reaction containing only the *C*-named KEGG compounds is used in the FDR generated by AutoKEGGrec. This particular reaction, however, contains the compound *C*06136, which is, according to the KEGG compound annotation, a generic compound with the formula *C*_45_*H*_79_*N*_2_*O*_23_*R*, and for that reason has no mass. While assembling this important compound information, such compounds are identified and the reaction will be marked in the “RxnAttention” field with *“This reactions contains a generic compound or a compound without mass in KEGG”*, so that these omitted reactions are easy do identify. These compounds are stored within the Matlab structure for omitted data so that the user can quickly access the compounds in questions, identify the reason for omission, and decide if and how the user wants to implement the compound/reaction into the (curated) reconstruction. Note that the field “RxnAttention” will be found within the model for included reactions for careful inspection, shown in the next example, as well as within the omitted reactions.

There are examples of glycan molecules which do not have a *C* compound KEGG ID, e.g. in the reaction

$$ R07807: G01977 + C00001 \Leftrightarrow G13073 + C00124. $$

It is added to the FDR because of a lack of alternative reactions, and will be marked in the “rxnAttention” annotation field. We encourage users to carefully inspect reactions with this tag that stay within the reconstruction. Additionally, we note that some glycan compounds do not have and *exact*_*mass* or *mol*_*mass* listed in KEGG, but only *mass*. However since there is a specific mass given which allows the user to manually check the reactions for mass consistency, AutoKEGGRec includes these compounds.

AutoKEGGRec has no intrinsic limit on the number of query organisms, all 65 *E. coli* strains stored in KEGG by their KEGG organism ID could be joined into one consolidated model, offering the user the union of the *E. coli* reaction networks available in KEGG along with corresponding gene links. Even unrelated organisms could be added to include their reactions, pathways, and related gene names.

Directly investigating the correspondence between the AutoKEGGRec FDRs and experimental data would make little sense, as the tool’s purpose is not to make a complete model capable of producing biomass. However, in order to give an idea of the amount of remaining work for any modeler employing the tool, we chose to compare the AutoKEGGRec FDR, created using the KEGG ID *eco*, to the published *E. coli* models iAF1260 [31] and iJO1366 [32], as well as a model made using ModelSeeds with default settings based on the stored data on *E. coli* K-12 MG1566 with the ID 511145.180. Comparing the quality of different GEMs is a complicated issue, not least in part due to differences in namespace, making alignment of different metabolic networks difficult.

Since two of the models are manually curated and the third is made from a template, they contain biomass and transport reactions. As AutoKEGGRec reconstructions do not, such reactions have been removed before comparison. That way the pure reaction networks can be compared to that of the KEGG-based reconstruction generated by AutoKEGGRec. The results can be seen in Table 3. Here, the models are listed with a direct comparison of the different chosen graph parameters, as well as other comparable parameters. Two separate numbers of reactions are given. They are marked with “BR” and “AR”, meaning before and after removal of transport reactions, respectively. The rest of the numbers are retrieved after removing such reactions. Some parameters are given for the metabolic network; these are the number of reactions in the reconstruction, the mean degree, and the mean shortest path for each model. Furthermore, the number of blocked metabolites is shown, as well as the number of metabolites occurring in exactly two reactions with the same sign in the S-matrix, is shown. As the transport reactions have been removed, a blocked metabolite is here defined as a metabolite occurring in exactly one reaction. Combined with the number of metabolites occurring in exactly two reactions, having the same sign in the S-matrix on both of them, they summarize the amount of potentially blocked metabolites. Additionally, the number of genes within each reconstruction, and, if possible due to namespace, the overlap with the AutoKEGGRec reconstruction in terms of number of genes are given. Summarizing the different properties of the models, it seems that *eco*, the FDR generated in AutoKEGGRec, is of comparable quality to the manually curated models iAF1260 and iJO1366. This speaks to the quality of KEGG, as well as FDRs generated using AutoKEGGRec.

**Table 3:** Comparison of *eco*, the FDR generated by AutoKEGGRec, with iAF1260 [31], iJO1366 [32], and ModelSeed model 511145.180 generated with default settings

Comparison	*eco*	*iAF1260*	*iJO1366*	*511145.180*
# of reactions BR	1224	2382	2583	1635
# of metabolites BR	1185	1668	1805	1573
# of reactions AR	1224	2081	2256	1510
# of metabolites AR	1185	1668	1805	1573
network mean degree	4.203	4.727	4.765	4.527
network mean shortest path	4.470	4.549	4.493	3.860
# of blocked metabolites	519	369	390	746
# of same-sign-metabolites	160	89	84	136
# of genes in model	1263	1260	1366	1139
# of shared genes with *eco*	1263	927	981	794

We removed all transport, biomass, and ATP maintenance reactions from iAF1260, iJO1366 and 511145.180. Except for reported values marked “BR” (before removal) and “AR” (after removal), results are for the reduced models. Definition of “blocked metabolites” and “same-sign-metabolites” is provided in the main text

## Discussion

AutoKEGGRec is an easy-to-use tool for the rapid assembly of first-draft reconstructions for genome-scale metabolic modeling including as much data as possible. While there exists a selection of tools and toolkits that aid in the assembly and refinement of first-draft GEMs, these tend to be stand-alone and/or online, both of which, especially in combination with small development teams in temporary academic positions, may cause any number of issues due to lack of support. These tools mostly create single-organism FDRs, as most are not designed for or allow the creation of community or generic/consolidated models.

Any automatic reconstruction necessitates steps such as gap-filling, mass and charge balance for reactions, reaction reversibility corrections, and adjustment of exchange, ATP maintenance and biomass reactions. These steps being mostly automated might appear as a convenience, but it is important to note that these tools often make implicit assumptions in the course of the reconstruction work without marking them as such. This is done in order to achieve functional models, i.e. models that are capable of producing the constituents of a biomass reaction, but, importantly, there are usually many ways of achieving this, and which way is chosen matters for the correctness and quality of the model, making control and traceability of the process an important issue. Many such educated guesses are based on template models or common knowledge, such as the inclusion of seemingly universally essential cofactors [33] in the biomass function. However, the way in which this information is retrieved and assembled is often not completely transparent, leaving important details of the model’s workings outside of a user’s control or awareness. Any resulting FDR may therefore contain misleading information, which is further propagated in reconstruction projects.

Biomass reactions in particular are known to be fickle with regards to cofactor coefficients [34]. This may lead to false growth predictions with regards to media and knockouts when naively propagated from skeleton models. Therefore, the manual curation of all reactions, and especially the biomass reaction, is still the recommended approach for most applications, and the added value of including template features such as biomass functions is debatable.

To varying degrees, these published tools automate the generation of first draft reconstructions, and greatly facilitate the reconstruction of GEMs. AutoKEGGRec, however, is not intended to automatically generate a first draft model with all requisite transport reactions and a biomass function. Since all information is based only on KEGG, every detail is eminently traceable, which may be of particular interest to modelers, as many “new” models are based on previous models, and may therefore inherit previous poor annotations and assumptions. The intent is to generate an unbiased and clean FDR of the metabolic network based only on the genome annotation in KEGG.

It is implemented as a Matlab function fully integrated with the COBRA toolbox, with its open, community-maintained and state-of-the-art suite of tools for model curation and manipulation. Additionally, AutoKEGGRec contains the complete annotation for all reactions and compounds offered by KEGG, putting more information right at the modeler’s fingertips than what is offered by most other comparable reconstruction tools.

In its rapid assembly of a draft reconstruction, AutoKEGGRec mostly covers the currently automatable parts of the first stage in the protocol to generate high-quality genome-scale metabolic reconstructions [35]. Additionally, it allows not only the creation of an FDR for a single organism, but also consolidated and community FDRs. These features set AutoKEGGRec apart from published methods. Consequently, it is a valuable tool for the current-day practitioner of genome-scale metabolic modeling and reconstruction.

While the reconstruction of microbial consortia remains an immature method, AutoKEGGRec can help speed up the process. Further work in assembling the reconstruction is still expected to rise steadily with the number of organisms, as not only does each separate organism’s metabolic network needs to be curated, but also the interactions between the different networks and their global effect on the environment. However, due to the chosen naming convention for compartments within the community models, transport reactions can easily be added simultaneously for multiple organisms if necessary.

## Conclusion

Here we present AutoKEGGRec, a fast tool to be used within the COBRA toolbox in Matlab that is able to create single organism, community, and consolidated first draft reconstructions based on the KEGG database. Unlike most other available tools, AutoKEGGRec is designed from the ground up to allow for community and consolidated metabolic network reconstructions. Also, being based only on data from KEGG, it has a clear and transparent data ancestry.

AutoKEGGRec does not provide a fully functioning model since elements such as a biomass function, ATP maintenance function, and most transport reactions for the given organism(s) are not included. The addition of these elements would require estimations and guesswork beyond the data available in KEGG at present. However, AutoKEGGRec does provide a very well annotated first draft reconstruction based on all data available in KEGG. As with reconstructed networks generated with any software package, the user should perform additional experiments, and add biomass function(s), ATP maintenance, and relevant import reactions to further enhance the reconstruction; all tasks which may be performed using existing functions in the COBRA toolbox. Consequently, AutoKEGGRec fills an important niche for the modeling community with its reliable and rapid generation of well annotated single, community, and consolidated FDRs within the COBRA toolbox environment based only on the clear, high-quality, and freely accessible data in KEGG.

## Availability and requirements

**Project name:** AutoKEGGRec

**Operating system(s):** Any which can run Matlab

**Programming language:** Matlab

**Other requirements:** Matlab, COBRA Toolbox version 3.0 or newer

**License:** Matlab

**Any restrictions to use by non-academics:** Licence needed for Matlab

An internet connection is required.

**Availability:** AutoKEGGRec will be submitted to be part of the COBRA toolbox. Additionally, the software is included as supplementary file, and is also freely available for download at https://www.ntnu.edu/almaaslab and https://github.com/emikar/AutoKEGGRec. AutoKEGGRec was developed and tested on Matlab (versions 2017a, 2017b and 2018a) and the COBRA toolbox v3.0.

## Additional files

Additional file 1 User manual for AutoKEGGRec. (PDF 5258 kb)

Additional file 2 The Matlab file for AutoKEGGRec. (M 83 kb)

## Abbreviations

COBRA: COnstraint-Based Reconstruction and Analysis
EC number: Enzyme Commission number
FDR: First Draft Reconstruction
GEMs: Genome-Scale Metabolic modelS
GPR: Gene Protein Relation
KEGG: Kyoto Encyclopedia of Genes and Genomes
OR: Organisms-Reactions
ORG: Organisms-Reactions-Genes
