# Supplementary material for: Automated generation of genome-scale metabolic draft reconstructions based on KEGG
Source: BMC Bioinformatics. 2018 Dec 4;19:467. doi: 10.1186/s12859-018-2472-z (PMC6280343; doi:10.1186/s12859-018-2472-z)
Supplement: Supplementary file 1 — User manual for AutoKEGGRec. (PDF 5258 kb) [file 12859_2018_2472_MOESM1_ESM.pdf]

AutoKEGGRec Manual

Emil Karlsen, Christian Schulz and Eivind Almaas

September 11, 2018

Contents

1 AutoKEGGRec **2**

2 Installation and requirements **2**

3 Usage **2**

3.1 Optional flags . . . 3
3.1.1 ConsolidatedRec . . . 4
3.1.2 SingleRecs . . . 5
3.1.3 CommunityRec . . . 5
3.1.4 writeSBML . . . 6
3.1.5 OmittedData . . . 7
3.1.6 OrgRxnGen . . . 10
3.1.7 DisconnectedReactions . . . 12
3.1.8 GenePlot . . . 13
3.1.9 Histogram . . . 14
3.1.10 General statements concerning optional inputs . . . 14
3.2 KEGG annotation and the ATTENTION field . . . 15
3.3 Authors' recommendation of usage . . . 15

4 Possible curation steps following execution of AutoKEGGRec **16**

4.1 General steps . . . 16
4.2 Community reconstruction refinement . . . 17

1 AutoKEGGRec

This Matlab function rapidly assembles first-draft reconstructions (FDRs) from KEGG based on KEGG organism IDs. See associated paper by E. Karlsen, C. Schulz, and E. Almaas, "Automated generation of genome-scale metabolic draft reconstructions based on KEGG".

2 Installation and requirements

AutoKEGGRec is a pipeline developed in Matlab 2017b and is designed to be a part of the COBRA toolbox. The requirements are:

- A stable internet connection
- A personal computer running Matlab
- A functioning installation of the COBRA toolbox v.3

The AutoKEGGRec.m file should be copied into a folder included in the Matlab search path, e.g. by use of the Matlab *pathtool* command. If everything is installed and working correctly, the commands *initCobraToolbox* and *AutoKEGGRec* [1] should work and should auto-complete themselves by using the TAB key.

3 Usage

The function takes one mandatory input and a number of optional inputs:

```
outputStruct =
    AutoKEGGRec(KEGG_organism_IDs, varargin)
```

The variable *KEGG_organism_IDs* is a string array where the elements consist of either 3- or 4-letter KEGG ID organism codes, or six letter code starting with T; e.g. $T00007$ and *eco* are both KEGG organism IDs for *Escherichia coli* K-12 MG1655.

Example input, using the five *E. coli* K-12 strains available as KEGG organism IDs (09/2018):

```
outputStruct =
    AutoKEGGRec(["eco","ecj","ecd","ebw","ecok"])
```

This function call generates an output structure containing the **consolidated model** based on the five K-12 strains. This is the default output in the

[1] **Note:** The code uses the Matlab command *parfor* to download the reactions and compounds from KEGG in parallel. Because of that, the parallel pool is started at the beginning of the code. By default, the normal local parallel pool is used. You can change the number of cores and threads by changing the parallel preferences in Matlab.

case that no further input options are given.

Optional flags can be specified as a list of keywords after the organism IDs. Examples of recommended usage of the pipeline is given below.

3.1 Optional flags

The nine optional inputs (flags) to use within AutoKEGGRec are:

1. 'ConsolidatedRec'
2. 'SingleRecs'
3. 'CommunityRec'
4. 'writeSBML'
5. 'OmittedData'
6. 'OrgRxnGen'
7. 'DisconnectedReactions'
8. 'GenePlot'
9. 'Histogram'

They can be added to the function call as (e.g. for the above mentioned function):

```
outputStruct =
    AutoKEGGRec(["eco","ecj","ecd","ebw","ecok"],
    'CommunityRec', 'OrgRxnGen', 'GenePlot', 'Histogram',
    'OmittedData')
```

In this example, the options would add the community first draft reconstruction of the five *E. coli* K-12 strains to the output Matlab structure named *outputStruct*. That structure would also contain the Organisms-Reactions-Genes matrix and a specification of the omitted reactions and compounds including reasoning for omitting them. Furthermore, two Matlab plots would appear, showing the gene plot and a histogram.

In the following part, the different options will be explained, including screenshots from Matlab 2017b and figures of the output. The three commands generating reconstructions, *ConsolidatedRec*, *SingleRecs* and *CommunityRec*, are explained first.

As AutoKEGGRec is running, some information will be shown in the Matlab command window, keeping the user updated on its progress. Note that AutoKEGGRec takes some time to access all the KEGG-based information and annotation available. For each reaction and compound, the annotation is stored within the FDR, using the KEGG categories if no COBRA supported field is available.

During determination of allowed compounds, the compound field "EXACT_MASS" and the glycan field "MASS" are summarized to into the field "MASS". This does not affect the compound field "MOL_WEIGHT".

3.1.1 ConsolidatedRec

Usage:

```
outputStruct =
    AutoKEGGRec(["eco","ecj","ecd","ebw","ecok"],
     'ConsolidatedRec')
```

or

```
outputStruct =
    AutoKEGGRec(["eco","ecj","ecd","ebw","ecok"])
```

This command prompts AutoKEGGRec to generate a consolidated first draft reconstruction for the query organisms and provides them as a Matlab COBRA model structure. It can be produced using the 'ConsolidatedRec' option or no option at all since this is the default output. The FDR will contain every reaction in which at least one of the query organisms in KEGG is present. An example can be seen in Fig. 1, where we have visualized the output metabolic network produced by AutoKEGGRec for the *E. coli* K-12 strains, which have the KEGG organism IDs *eco*, *ecj*, *ecd*, *ebw*, and *ecok*.

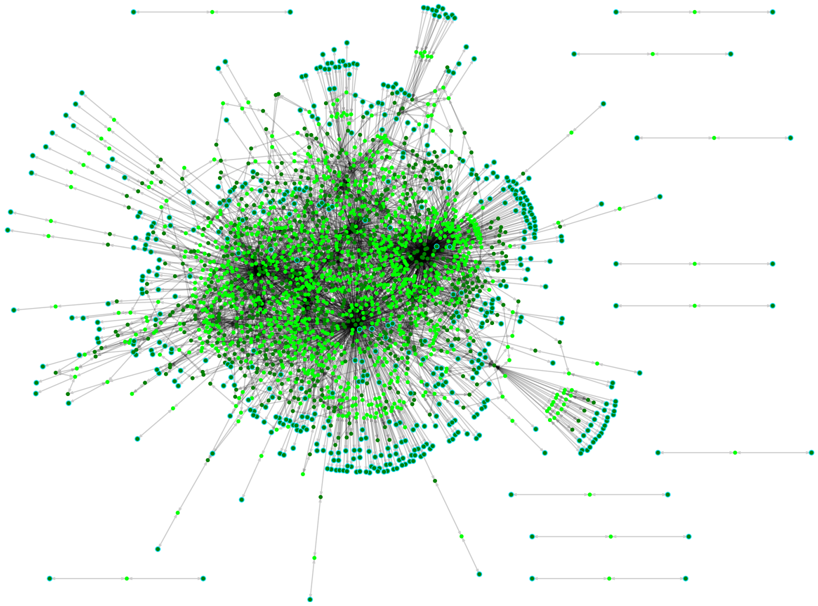

Figure 1: The consolidated reconstruction network based on the five *E. coli* K-12 strains, based on an SBML file written using the *writeSBML* option.

The consolidated reconstruction contains no gene-protein-reaction rules since different organisms will have different gene names, and possibly different numbers of genes, corresponding to the same reaction. The Matlab structures, however, include all possible KEGG based annotation for reactions and compounds.

3.1.2 SingleRecs

Usage:

```
outputStruct =
    AutoKEGGRec(["eco","ecj","ecd","ebw","ecok"],
    'SingleRecs')
```

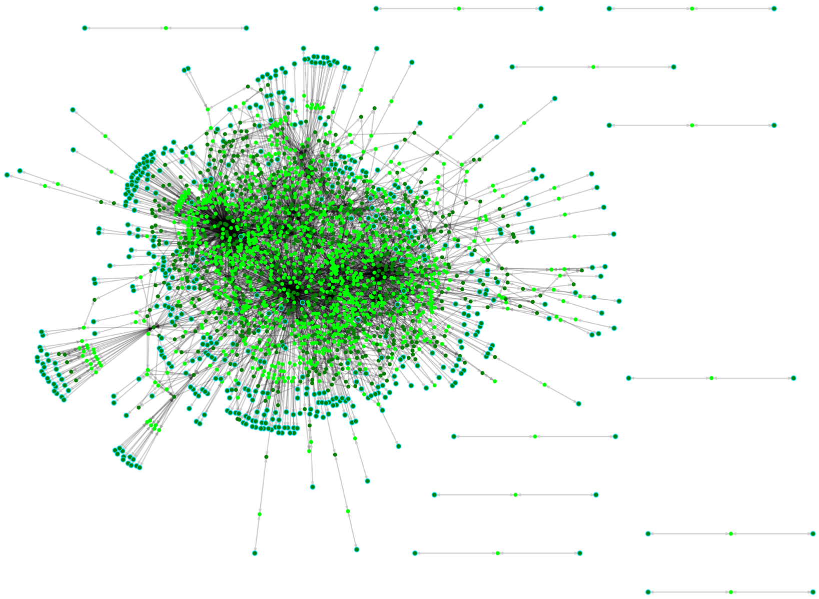

Figure 2: The reconstruction network of *E. coli* K-12 MG1655 using AutoKEGGRec for KEGG organism ID *eco*, based on an SBML file written using the *writeSBML* option.

Using this option, AutoKEGGRec creates single first draft reconstructions for each of the listed query organisms, each of which is stored by the organism KEGG ID within the AutoKEGGRec output. In Fig. 2, we have visualized the output metabolic network for *E. coli* K-12 MG1655 with KEGG organism ID *eco*. Each reconstruction contains the reactions present in the KEGG organism and the gene-protein-reaction rules, including all possible annotations for reactions and compounds based on the KEGG database.

3.1.3 CommunityRec

Usage:

```
outputStruct =
    AutoKEGGRec(["eco","ecj","ecd","ebw","ecok"],
    'CommunityRec')
```

This option creates a community first draft reconstruction based on the given organisms. The different query organisms are placed in separate compartments, specified by the organism KEGG ID as follows:

$$R00004\_eco[c] : C00013\_eco[c] + C00001\_eco[c] \quad \Leftrightarrow \quad 2C00009\_eco[c]$$

Consequently, each organism with their respective reactions and compounds is easy to identify. Each organism may have sub-compartments as well, as indicated by the $[c]$ (cytosol). Note that the single organisms are not connected. The FDR is purely based on the information in KEGG and no transport reactions sharing a common compound is present because of the naming of the compounds. For exchange reactions, typically $[e]$ is used to signify extra-cellular reactions/metabolites, which can be easily implemented with a Matlab program using a for-loop.

Each of the separate organism reconstructions has gene-protein-reaction rules included within the reconstruction, and an example of a community reconstruction can be seen in Fig. 3.

The FDR is stored within the AutoKEGGRec output as a COBRA model. Since it contains every reaction of every organism, and thereby e.g. central reactions several times, we decided to not include the most comprehensive AutoKEGGRec annotations possible. They are however stored within the other structures given by using the options *ConsolidatedRec* or *SingleRecs*. Therefore we recommend not only generating a community FDR using AutoKEGGRec but also a consolidated model of the given input organisms.

3.1.4 writeSBML

```
outputStruct =
    AutoKEGGRec(["eco","ecj","ecd","ebw","ecok"],
    'ConsolidatedRec', 'writeSBML')
```

NB: This option requires a reconstruction to be built. Refer to one of the options *ConsolidatedRec*, *SingleRec* or *SommunityRec*. If called without an explicit option to generate a reconstruction, an error message will appear suggesting the user generate a reconstruction.

This option calls the COBRA function *writeCbModel* after creating the requested reconstruction(s) to write the model as SBML file. The file will be saved in the "Current Folder" and automatically given a name based on the orgnaism ID(s) and current time and date, according to the following format: "*ExampleRec*_YYYY.MM.DD_hh.mm.xml" (for example *ConsolidatedRec*_2018.04.10_11.43.xml for a consolidated reconstruction generated 11:43 on the 10th of April 2018). In case that several SBML files are to be written, a Matlab window pops up showing the progress, where the progress of the loading bar is based on the number of <u>reactions</u>, not reconstructions, in order to give the user an impression of progress in terms of required work.

Keep in mind that some fields added to the reconstructions by AutoKEGGRec are not supported by the COBRA *writeCbModel* function, and so saving

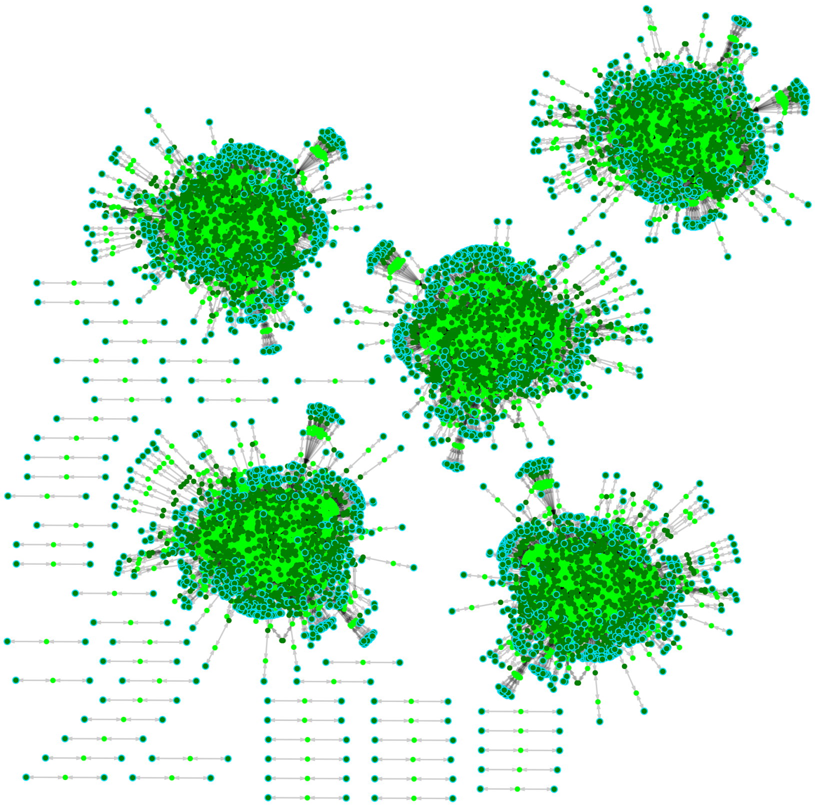

Figure 3: The community FDR network for the five *E. coli* K-12 strains. Since no transport reactions are added, the separate compartments (different organisms) are not connected.

the output variable as a Matlab structure is recommended if the user wants to keep some of the additional data, such as the omitted reactions (see Sec. 3.1.5) or the OrgRxnGen matrix (see Sec. 3.1.6). Other valuable annotations based on the KEGG data, such as the mass of compounds, will also be lost if not stored with a *.mat* file.

3.1.5 OmittedData

Usage:

```
outputStruct =
    AutoKEGGRec(["eco","ecj","ecd","ebw","ecok"],
    'OmittedData')
```

This optional input can be used to analyze the KEGG reactions rejected by AutoKEGGRec, and gives a sub-structure in the AutoKEGGRec output

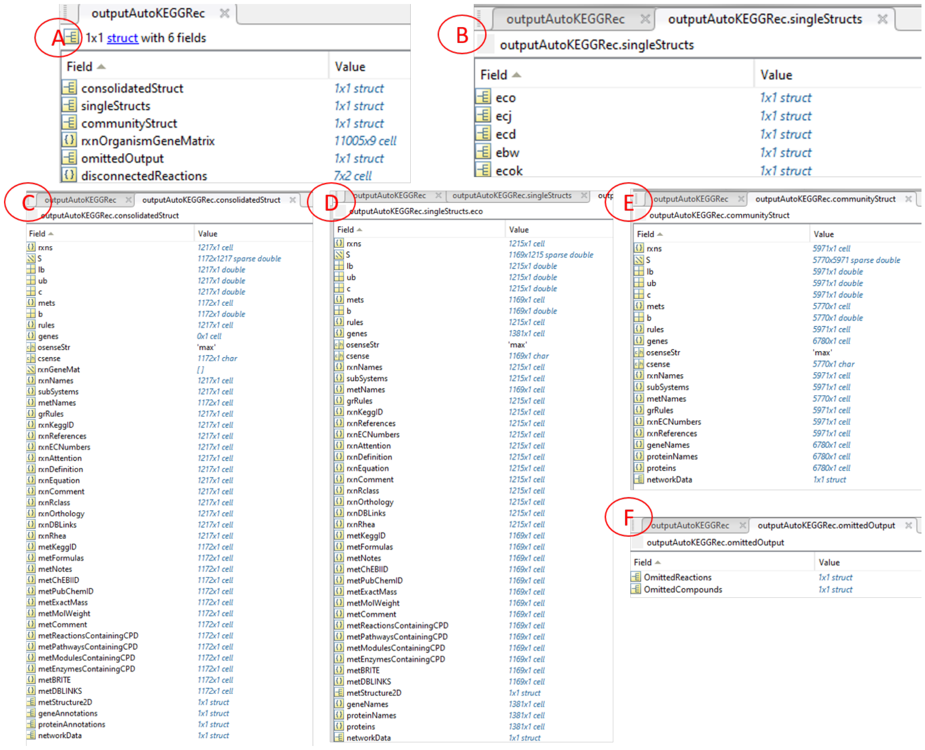

Figure 4: Screenshots of the AutoKEGGRec output structure of the FDRs for the five *E. coli* K-12 strains that a user can expect. In (A), the structure of the output variable (*outputStruct* in the examples) is shown. All the single first draft reconstructions are shown in (B), whereas the single first draft reconstruction *eco* is shown in (D), the consolidated first draft reconstruction and the community first draft reconstruction are shown in (C) and (E), respectively. In (F) we present the first layer of the omitted data, which is further presented in Fig. 5 and described in Sec. 3.1.5.

structure. Within that sub-structure are two fields, one which contains the omitted compounds and two, which contains the omitted reactions (refer to Fig. 4 (F) and Fig. 5 (A) and (B)).

Reactions omitted by AutoKEGGRec (e.g. polymerization reactions, general reactions, reactions with generic compounds, etc.) are stored here with their annotations, making these reactions available to the user. The reactions, as well as the compounds, are stored within a sub-structure in the omitted output and sorted, and are easily accessible by their KEGG IDs. Within the KEGG annotation the original (i.e. not cleaned) reaction and any further data available on the reaction in KEGG (example shown in Fig. 5 (D)) can be seen. Furthermore, the field "Attention" is added, including the the reason the reaction was omitted. This allows the user to quickly determine whether and how

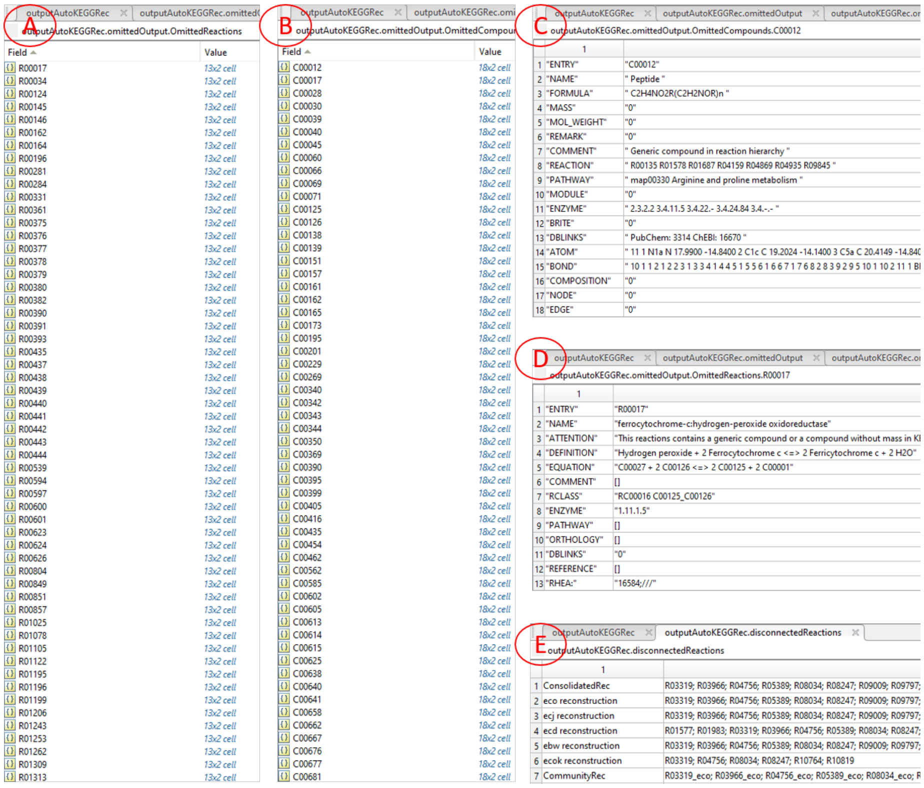

Figure 5: Screenshots of the output structure in Matlab for the five *E. coli* K-12 strains. The fields within the omittedOutput using the options *omittedData* are shown in Fig 4 (F). In (A) and (B), the omitted reactions and compounds, respectively, are presented sorted by their KEGG IDs, each being a Matlab cell. These cells are shown in (C) and (D) for the containing annotation based on KEGG for the compound and reaction, respectively. Note the added ”Attention” field in (D), which contains the reason for omission. A corresponding field for the compounds is not added, since the only criterion to omit a compound is $mass = 0$. In (E) the content of the Matlab cell ”disconnectedReactions” (see Fig. 4 (A)) using the AutoKEGGRec option *DisconnectedReactions* is presented. Here, in a summary for each generated reconstruction, all reactions that are not connected to the giant component (refer to the network figures in Figs 1, 2 and 3) are summarized to allow the user easy access to these reactions.

to implement such reaction during the curation process.

The KEGG compounds are assessed within AutoKEGGRec and added to the *OmittedData* if their mass is 0. Note that AutoKEGGRec uses the metMass field to include the mass of specific glycan compounds as well as the exact_mass of KEGG compounds; they are also summarized in the field ”MASS” in the

omitted output where applicable, refer to Fig. 5 (C).

Within the listed the KEGG IDs of omitted compounds, the whole KEGG annotation of each compound is stored as a Matlab cell (Fig. 5 C). The user can access these fields, retrieve all stored KEGG annotations to the compound in question directly to decide whether and how to implement that compound and the corresponding reaction.

All strings within the specific fields in the Matlab struct are space-separated fields, which allows the user quick access and possibilities for simple search in e.g. the reactions a specific compound participates in.

3.1.6 OrgRxnGen

Usage:

```
outputStruct =
    AutoKEGGRec(["eco","ecj","ecd","ebw","ecok"],
    'OrgRxnGen')
```

This option instructs AutoKEGGRec to create the "Organisms-Reactions-Genes matrix" (example seen in Fig. 6) and store it within the output structure. Within this matrix, all available KEGG reaction IDs are stored (first column). Each following column represents one of the requested organisms, and if a certain reaction is present within an organism, the gene name(s) for that reaction will be in this field. In case of several genes they are listed and separated by a bar ("|"), which represents the "OR" relationship within the COBRA toolbox. In reality, the relationship between genes pertaining to a given reaction may be any combination of "AND"/"OR" relationships, but this information is currently not available in the KEGG database. This matrix is also the basis for generating the reconstructions and implementing the gene-reaction-relationship.

The last three columns contain some summary data for each reaction, which may be of help during analysis:

- Sum
- Total
- Genes

The "Sum" column (third to last column) gives the sum of how many organisms contain genes related to a given reaction.
The "Total" column (second to last column) gives the sum divided by the number of organisms, and therefore describes the fraction of organisms which has the reaction in their metabolic network according to KEGG.
The "Genes" column (last column) states the number of genes within the organisms related to this reaction. In case of different numbers of genes for the different organisms for a given reaction, the values are comma separated.

This matrix may be useful for certain kinds of analysis on organisms stored in KEGG, such as this short Matlab code snippet to select all the reactions that have different number of genes related to them (example output seen in Fig. 7):

output | output.rxnOrganismGeneMatrix

output.rxnOrganismGeneMatrix

	1	2	3	4	5	6	7	8	9
1	'KEGG ID'	'eco'	'ecj'	'ecd'	'ebw'	'ecok'	'Sum'	'Total'	'Genes'
2	'R00004'	''	''	''	''	''	'0'	'0'	'0'
3	'R00006'	''	''	''	''	''	'0'	'0'	'0'
4	'R00009'	'b4226'	'JW4185'	'ECDH10B_...	'BWG_3936'	'ECMDS42_...	'5'	'1'	'1'
5	'R00010'	''	''	''	''	''	'0'	'0'	'0'
6	'R00013'	'b0078 | b3...	'JW0077 | J...	'ECDH10B_...	'BWG_0073...	'ECMDS42_...	'5'	'1'	'3, 5'
7	'R00014'	''	''	''	''	''	'0'	'0'	'0'
8	'R00017'	'b1732 | b3...	'JW1721 | J...	'ECDH10B_...	'BWG_1545...	'ECMDS42_...	'5'	'1'	'2'
9	'R00022'	'b1197 | b3...	'JW3487 | J...	'ECDH10B_...	'BWG_3208...	'ECMDS42_...	'5'	'1'	'2'
10	'R00026'	''	''	''	''	''	'0'	'0'	'0'
11	'R00028'	''	''	''	''	''	'0'	'0'	'0'
12	'R00034'	'b0507'	'JW0495'	'ECDH10B_...	'BWG_0384'	'ECMDS42_...	'5'	'1'	'1'
13	'R00036'	'b0114 | b0...	'JW0110 | J...	'ECDH10B_...	'BWG_0073...	'ECMDS42_...	'5'	'1'	'4, 6'
14	'R00066'	''	''	''	''	''	'0'	'0'	'0'
15	'R00078'	'b3518'	'JW3486'	'ECDH10B_...	'BWG_3207'	'ECMDS42_...	'5'	'1'	'1'
16	'R00084'	''	''	''	''	''	'0'	'0'	'0'
17	'R00086'	''	''	''	''	''	'0'	'0'	'0'
18	'R00087'	''	''	''	''	''	'0'	'0'	'0'
19	'R00089'	'b1107'	'JW1093'	'ECDH10B_...	'BWG_0955'	'ECMDS42_...	'5'	'1'	'1'
20	'R00093'	''	''	''	''	''	'0'	'0'	'0'
21	'R00094'	''	''	''	''	''	'0'	'0'	'0'
22	'R00097'	''	''	''	''	''	'0'	'0'	'0'
23	'R00103'	'b2132'	'JW2120'	'ECDH10B_...	'BWG_1916'	'ECMDS42_...	'5'	'1'	'1'
24	'R00104'	''	''	''	''	''	'0'	'0'	'0'
25	'R00112'	'b0403'	'JW0393'	'ECDH10B_...	'BWG_0285'	'ECMDS42_...	'5'	'1'	'1'
26	'R00114'	''	''	''	''	''	'0'	'0'	'0'
27	'R00115'	''	''	''	''	''	'0'	'0'	'0'
28	'R00124'	''	''	''	''	''	'0'	'0'	'0'
29	'R00125'	''	''	''	''	''	'0'	'0'	'0'
30	'R00127'	'b0708'	'JW0698'	'ECDH10B_...	'BWG_0567'	'ECMDS42_...	'5'	'1'	'1'
31	'R00130'	'b0369'	'JW0361'	'ECDH10B_...	'BWG_0256'	'ECMDS42_...	'5'	'1'	'1'

Figure 6: Screenshot of the "Organisms-Reactions-Genes matrix" Matlab field of the first draft reconstructions of the five *E. coli* K-12 strains provided by AutoKEGGRec.

```
lines =
    false(length(outputStruct.rxnOrganismGeneMatrix));
lines(1) = true;
for line=1:length(lines)
    lineContents =
        outputStruct.rxnOrganismGeneMatrix(line,:);
    if contains(string(lineContents(end)),",")
        lines(line) = true;
    end
end
selectedLines =
    string(outputStruct.rxnOrganismGeneMatrix(lines,:))
```

The potential of generic reconstructed models for a species is demonstrated

selectedLines

9x6 string

	1	2	3	4	5	6
1	KEGG ID	eco	ecj	Sum	Total	Genes
2	R02059	b0677	JW0663 | JW5527	2	1	1, 2
3	R04111	b1621	JW1613 | JW3659	2	1	1, 2
4	R05168	b0677	JW0663 | JW5527	2	1	1, 2
5	R05747	b3503 | b2495	JW3470 | JW2480 | JW2619	2	1	2, 3
6	R06236	b1621	JW1613 | JW3659	2	1	1, 2
7	R11325	b0734 | b0979 | b0733 | b0978 | b4592 | b4515	JW0723 | JW0961 | JW0722 | JW0960 | JW0724	2	1	5, 6
8	R11885	b0734 | b0979 | b0733 | b0978 | b4592 | b4515	JW0723 | JW0961 | JW0722 | JW0960 | JW0724	2	1	5, 6
9	R11886	b0734 | b0979 | b0733 | b0978 | b4592 | b4515	JW0723 | JW0961 | JW0722 | JW0960 | JW0724	2	1	5, 6

Figure 7: Screenshot of the output for shown Matlab code used on the OrgRxnGen output based on two of the five *E. coli* K-12 strains. It shows the first part of all reactions that are encoded by a different number of genes within the two organisms.

in the human RECON model: Many different cell types can be stored within a single model, making the data compact and manageable. Due to many similar core reactions, which are used by all the different cells, this type of model gives a detailed yet broad overview of the human cell's metabolic network. In the case of *E. coli*, more specifically the five *E. coli* K-12 strains, it gives a detailed overview of the reaction network; the ORG matrix in combination with the different plots describe similarities and differences among the *E. coli* K-12 strains.

3.1.7 DisconnectedReactions

Usage:

```
outputStruct =
    AutoKEGGRec(["eco","ecj","ecd","ebw","ecok"],
    'ConsolidatedRec', 'DisconnectedReactions')
```

NB: This option requires a reconstruction to be built. Refer to one of the options *ConsolidatedRec*, *SingleRec* or *SommunityRec*. If called without an order to generate a reconstruction, an error message will appear, suggesting the user generate a reconstruction.

AutoKEGGRec creates a cell inside the output containing the KEGG reaction IDs for all the reactions that are disconnected from the giant component. It does not matter if the reactions are connected to each other; all reactions not connected to the giant component are listed her for every generated reconstruction. Example output is shown in Fig. 5 (E). AutoKEGGRec automatically creates a "networkData" entry for every reconstruction as an annotation field. The user can easily access the network information such as the number of reactions in the giant component and the sizes of the disconnected components, as well as directly identify disconnected reactions.

3.1.8 GenePlot

Usage:

```
outputStruct =
    AutoKEGGRec(["eco","ecj","ecd","ebw","ecok"],
    'GenePlot')
```

AutoKEGGRec creates a Matlab plot window containing the gene plot (example seen in Fig. 8). It can be saved, processed and adapted within the Matlab window. The plot shows the number of genes vs. the number of reactions for each of the requested organisms. By default, the Y-axis is in log-scale, but this can be changed within the Matlab plot window. In the figure the output for five *E. coli* K-12 strains provided as input to AutoKEGGRec using this flag is presented, resulting in the generation of five plots where each plot shows a bar-diagram of reaction associations per organism.

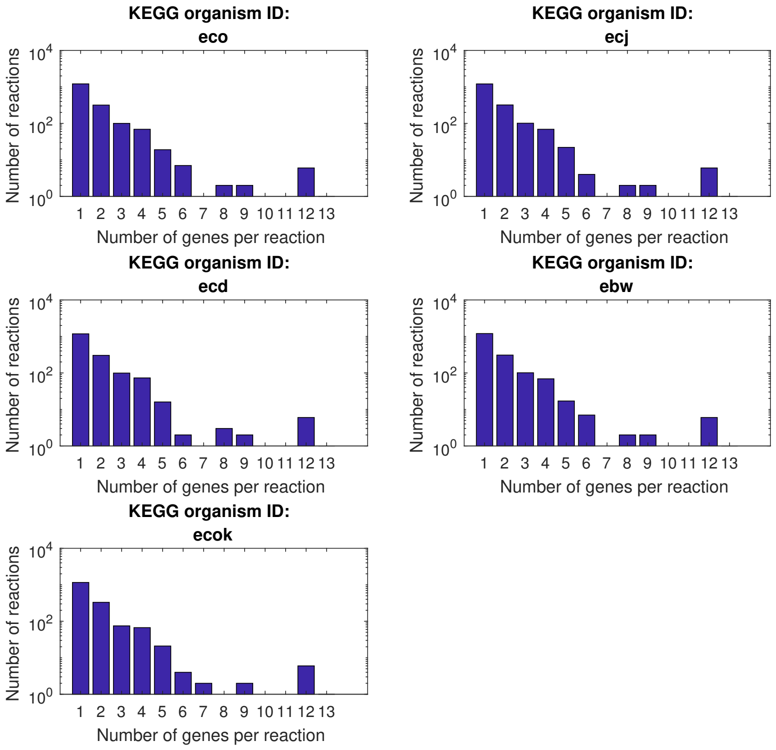

Figure 8: The Matlab plot for the five *E. coli* K-12 strains. The plot for each strain shows the number of genes vs. the number of reactions, which gives a quick overview of the number of reactions encoded by a specific number of genes.

3.1.9 Histogram

Usage:

```
outputStruct =
    AutoKEGGRec(["eco","ecj","ecd","ebw","ecok"],
    'Histogram')
```

AutoKEGGRec opens a Matlab plot window containing a histogram showing the number of organisms sharing a number of reactions (example seen in Fig. 9 for the five *E. coli* K-12 strains). The X-axis shows the number of organisms, starting at one and ending at the number of organisms, whereas the (by default logarithmic) Y-axis gives the number of reactions that occur in the particular number of organisms. The user can use this plot to get an idea of common reactions throughout the input organisms; it could help the user to identify the number of core/conserved reactions within the metabolic networks based on the organisms. In this example, i.e. the five *E. coli* K-12 strains in KEGG, most of the reactions are shared between the five organisms. Interestingly, there are some which occur only in one of the strains, as well as some that are common in three strains.

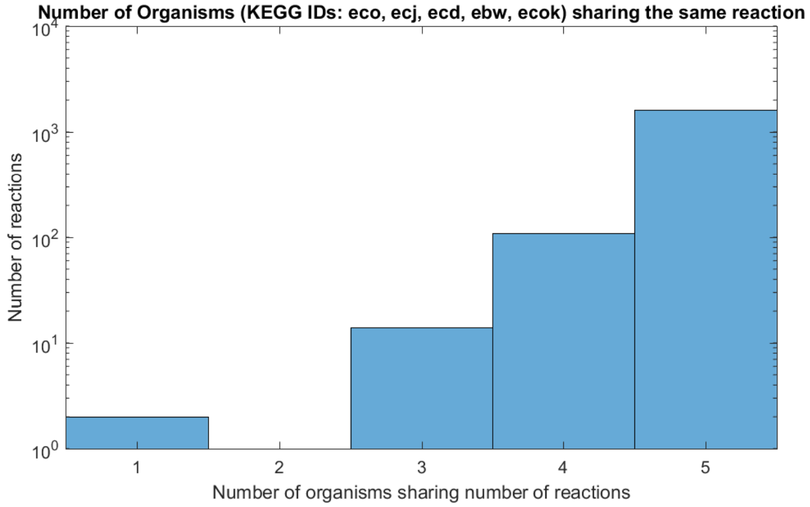

Figure 9: The histogram shows the number of organisms, here the five *E. coli* K-12 strains, vs. the number of reactions. The plot helps to identify the amount of core reactions shared within the input organism KEGG IDs.

3.1.10 General statements concerning optional inputs

To ensure that the options are correctly used, the options relying on model building are checked, as well as spelling of the options. There will be error messages stating what went wrong, and by referencing the help function or this

manual, typos can be rapidly identified. The order of the input options does not matter.

3.2 KEGG annotation and the ATTENTION field

All KEGG annotation is saved within the same fields to be seen in KEGG within the reconstructions as well as within the omitted output. Changes to the category names, however, do happen in case of fields supported by COBRA within the annotation of the reconstructions.

Additionally, AutoKEGGRec introduces a new field in reconstruction and omitted output, the "ATTENTION" field. Within the Reconstruction, AutoKEGGRec notes suspicious reactions for further inspection. The user may use that field to note and annotate curated reactions for the tractability of information. Within the omitted output, AutoKEGGRec notes in that field, why the reaction was rejected to be implemented (see Fig. 5 (D)). In case of e.g. the rejected reaction

$$R00164 : C00562 + C00001 \quad \Leftrightarrow \quad C00017 + C00009,$$

the user would find within the annotation field in the omitted output:

"This reactions contains a generic compound or a compound without mass in KEGG. Check the compounds carefully before adding reaction to model!",

which allows the user to quickly identify the reason. In this case, checking the omitted compound list (see Fig. 5 (B)) for any of such points to the reason: the compounds $C00562$ and $C00017$ ("phosphoprotein" and "protein", respectively) do not have a mass and can therefore not be mass balanced. The user can check all the compounds of the reaction and decide whether and how to implement them.

3.3 Authors' recommendation of usage

AutoKEGGRec is intended as a tool that generates various first-draft reconstructions and delivers them to the user along with some helpful support for the following manual curation to allow the creation of a high quality model. Because of this, AutoKEGGRec ships with several different options to optimize run-time for the purposes of the different users. This way, AutoKEGGRec can be used not only to create reconstructions, but also to explore the KEGG reaction universe for any number of given organisms and base any analysis on this information.

However, to generate first-draft reconstructions, the recommended set of input parameters is:

```
outputStruct = AutoKEGGRec("ORGANISM_IDs",
    'RECONSTRUCTION', 'OrgRxnGen', 'OmittedData',
    'DisconnectedReactions', 'GenePlot', 'Histogram')
```

With this command, AutoKEGGRec creates (up to several) reconstructions for the selected KEGG organism IDs and delivers supporting information to aid in further model curation. The user is advised to save the plots and the generated Matlab output structure as .mat file. This way, all necessary information is stored and can be retrieved quickly.

Additionally, using the ATTENTION field, users can implement comments during the curation process, which allows future user of the model to easy trace any kind of data and reasoning why such reaction is present within the model. Thorough annotation and comments highly improves the (re)usability of the model.

4 Possible curation steps following execution of AutoKEGGRec

4.1 General steps

The most important part of your reconstruction process comes after you are done using AutoKEGGRec. No matter where your first draft comes from, and what functionality it does or does not contain, manual curation is absolutely essential.

Drawing on the 96-step protocol for generating high-quality genome-scale metabolic reconstructions set forth by Thiele and Palsson in 2010, we here suggest what steps to take (without right of completeness) when generating your own genome-scale metabolic reconstruction:

1. Generating the Reconstruction using AutoKEGGRec
2. Adding and correcting exchange reactions (import reactions for cytosol). Adding exchange reactions can be done easily using addExchangeRxn()
3. Adding biomass and ATP maintenance functions and checking if the biomass compounds are producible. Adding reactions is simple using the COBRA function addReaction()
4. Checking mass and charge balance for reactions. For this you can use the COBRA function checkMassChargeBalance()
5. Checking reversibility of reactions using such databases as KEGG pathways, as well as the COBRA function using thermodynamics
6. Gap filling algorithms (COBRA fastGapFill())
7. Manual gap filling (COBRA gapFind())
8. Adding additional compartments and the corresponding reactions and transport reactions (COBRA addReaction(), addExchangeRxn())

9. Verify gene-protein-reaction association

10. Add demand and sink reactions (COBRA addSinkReaction(), addDemandReaction())

11. Checking dead ends and flux consistency (COBRA detectDeadEnds(), findFluxConsistentSubset(), fastLeakTest())

Repeat step 4 to 11 iteratively for different Biomass functions and/or environments. These steps are partly carried out by some tools/functions but they require active user involvement. Furthermore, in-between each step, which may contain several substeps (as there will likely be more than one transport reaction to be added), testing the model is absolutely necessary.

In the example presented here, most of the steps only usee Matlab COBRA Toolbox functions. Other alternatives also exist, e.g. COBRApy. Since the reconstructions contain all possible KEGG annotation, which includes for compounds e.g. PubChem IDs, it is easy to cross-reference the compounds to other databases as well, if other databases do not contain the KEGG IDs.

Many steps require **manual** curation to ensure high quality of the models, the tools and functions only support the modeller.

Implementing as much data for compounds and reactions as possible into the model may save future work, and has few drawbacks. It might allow for easier comparison of models and also additional uses, e.g. the masses of the compounds for further applications. In particular, including the masses allows for a fast and easy check on the mass balance of the reconstructions.

4.2 Community reconstruction refinement

Since AutoKEGGRec is designed to also generate communities based on organism KEGG IDs, the Authors want to highlight possible further steps after using AutoKEGGRec. A community reconstruction should be able to model interactions between the organisms contained in the community. Therefore interaction reactions are necessary.

As described in Section 3.1.3, all the reactions and compounds in the reconstruction follow the same pattern

$$R00004\_eco[c] : C00013\_eco[c] + C00001\_eco[c] \quad \Leftrightarrow \quad 2\ C00009\_eco[c],$$

which allows the user to edit several reactions at a time.

As shown in the example network in Fig 3.1.3, none of the organisms are connected. AutoKEGGRec only generates reconstructions based on the KEGG database, which includes few transport reactions to link the single organisms. However, a short script to add "standard uptakes" and linking the organisms for gap filling might ease the modeler's work as described in Section 4.1.
